# Recommendations of the Advisory Committee on Immunization Practices for Use of Hepatitis A Vaccine for Persons Experiencing Homelessness

**DOI:** 10.15585/mmwr.mm6806a6

**Published:** 2019-02-15

**Authors:** Mona Doshani, Mark Weng, Kelly L. Moore, José R. Romero, Noele P. Nelson

**Affiliations:** ^1^Division of Viral Hepatitis, National Center for HIV/AIDS, Viral Hepatitis, STD, and TB Prevention, CDC; ^2^Department of Health Policy, Vanderbilt University School of Medicine, Nashville, Tennessee; ^3^Pediatric Infectious Diseases Section, University of Arkansas for Medical Sciences and Arkansas Children’s Hospital, Little Rock, Arkansas.

Hepatitis A (HepA) vaccination is recommended routinely for children at age 12–23 months, for persons who are at increased risk for hepatitis A virus (HAV) infection, and for any person wishing to obtain immunity. Persons at increased risk for HAV infection include international travelers to areas with high or intermediate hepatitis A endemicity, men who have sex with men, users of injection and noninjection drugs, persons with chronic liver disease, person with clotting factor disorders, persons who work with HAV-infected primates or with HAV in a research laboratory setting, and persons who anticipate close contact with an international adoptee from a country of high or interme­diate endemicity (*1*–*3*). Persons experiencing homelessness are also at higher risk for HAV infection and severe infection-associated outcomes. On October 24, 2018, the Advisory Committee on Immunization Practices (ACIP)* recommended that all persons aged 1 year and older experiencing homelessness be routinely immunized against HAV. The ACIP Hepatitis Vaccines Work Group conducted a systematic review of the evidence for administering vaccine to persons experiencing homelessness, which included a set of criteria assessing the benefits and adverse events associated with vaccination. HepA vaccines are highly immunogenic, and >95% of immunocompetent adults develop protective antibody within 4 weeks of receipt of 1 dose of the vaccine (*1*). HAV infections are acquired primarily by the fecal-oral route by either person-to-person transmission or via ingestion of contaminated food or water. Among persons experiencing homelessness, effective implementation of alternative strategies to prevent exposure to HAV, such as strict hand hygiene, is difficult because of living conditions among persons in this population. Integrating routine HepA vaccination into health care services for persons experiencing homelessness can reduce the size of the at-risk population over time and thereby reduce the risk for large-scale outbreaks.

## Introduction

In 2017 in the United States, 1.42 million persons used an emergency shelter or transitional housing program at some point during the year (*4*). Estimates of homelessness are higher when unsheltered persons are considered. Some studies estimate that 2.3 million to 3.5 million persons experience homelessness each year (*5*), and persons of color are disproportionately affected (*4*,*5*). In 2017, on a single night, an estimated 553,742 persons experienced homelessness in the United States, approximately 35% of whom were in unsheltered locations (*4*). Although the number of persons experiencing homelessness has declined overall since 2007, the number of unsheltered persons experiencing homelessness in major cities has increased, and disparities remain (*4*). Persons experiencing homelessness are at 1.5 to 11.5 times the risk for mortality compared with the general population (*6*). Homelessness has been associated with substantial health inequalities, including shorter life expectancy; poor access to health care, resulting in delayed clinical presentation; higher morbidity; and greater use of acute hospital services, often for preventable conditions (*6*,*7*).

HAV infection is associated with poor sanitation and hygiene and is transmitted by the ingestion of contaminated food or water or by direct contact with an infectious person. Congregate living conditions, both within and outside shelters, increase the risk for disease transmission, which can result in outbreaks (*6*). Recent outbreaks with direct HAV transmission among persons reporting homelessness signal a shift in HAV infection epidemiology in the United States (*8*). During 2017, a total of 1,521 outbreak-associated HAV cases were reported from California, Kentucky, Michigan, and Utah, with 1,073 (71%) hospitalizations and 41 (3%) deaths; the majority of infections were among persons reporting homelessness or injection or noninjection drug use (*8*). The person-to-person HAV outbreaks involving persons who use drugs or persons experiencing homelessness are ongoing, and case counts and geographic dispersion increased substantially in 2018.^†^ As of October 12, 2018, approximately 7,000 outbreak-associated cases had been reported from 12 states (*8*).

Hepatitis A vaccines are critical to the prevention of HAV infection among persons experiencing homelessness. Detectable antibodies persist for at least 20 years after HepA vaccination in childhood (*9*), and antibodies persist for an estimated 40 years or longer based on mathematical modeling and anti-HAV kinetic studies (*9*). Although recommended as a 2-dose series, evidence of protection for up to 11 years exists for 1 dose of single-antigen vaccine (*10*); clinical and outbreak response experience suggests that lifelong protection is possible after 1 dose. Owing to limited access to health care and historically low rates of insurance coverage, the majority of adults who experience homelessness have low rates of immunization coverage with vaccines routinely recommended for adults. Community health centers provide preventive and primary health services to meet the specific needs of persons experiencing homelessness, including vaccination. Street or shelter-based interventions for targeted populations have been used as efficient methods for vaccinating persons experiencing homelessness during outbreaks (*11*). Thirty-six states and the District of Columbia have expanded Medicaid under the Affordable Care Act, providing an increase in coverage and access to care among persons experiencing homelessness; an estimated 77% had access to some form of insurance in 2017 (*12*).

This report provides recommendations for use of HepA vaccine among persons experiencing homelessness and updates previous ACIP recommendations for HepA vaccine that did not include homelessness as an indication for use of HepA vaccine for preexposure protection against HAV infection (*1*).

## Methods

During February 2018–October 2018, the ACIP Hepatitis Vaccines Work Group^§^ held monthly conference calls to review and discuss relevant scientific evidence^¶^ supporting inclusion of homelessness as an indication for HepA vaccine. The work group evaluated the quality of evidence related to the benefits and harms of administering HepA vaccine to persons experiencing homelessness using the Grading of Recommendations Assessment, Development and Evaluation (GRADE) framework (https://www.cdc.gov/vaccines/acip/recs/grade/table-refs.html).

At the October 2018 ACIP meeting, the following proposed recommendations were presented to the committee: all persons aged 1 year and older experiencing homelessness should be routinely immunized against hepatitis A. After a period for public comment, the recommendations were approved unanimously by the voting ACIP members.**

## Summary of Key Findings

**Homelessness as an indication for hepatitis A vaccination.** Little is known about HAV seroprevalence among homeless populations in the United States. Review of the literature found few studies that considered homelessness as an independent risk factor. Based on the evidence to recommendations framework, other considerations were assessed, such as recent HAV outbreaks (*8*), HAV-related hospitalizations and deaths, treatment costs for liver transplants, and the benefits and costs associated with HepA vaccination (https://www.cdc.gov/vaccines/acip/recs/grade/table-refs.html). These studies concluded that the benefits of vaccinating persons experiencing homelessness were substantial and the cost and risk of vaccinating persons experiencing homelessness is much lower than the risk of not vaccinating.

The clinical trial and observational studies that were included in the GRADE review had several limitations, and some did not report any quantitative data. The studies had limitations in design and execution. No comparison/control groups were present, and there was a serious risk of bias, inconsistency, indirectness, and imprecision. Only one study was found with vaccine immunogenicity data among the homeless population, and it reported on a non-U.S. population.

**GRADE quality of evidence summary for HepA vaccine among homeless persons.** The evidence assessing benefits and harms of administering HepA vaccine to prevent HAV infection in persons experiencing homelessness was determined to be GRADE evidence type 4 (i.e., evidence from clinical experience and observations, observational studies with important limitations, or randomized controlled trials with several major limitations) for benefits and for harms. The balance of consequences for the evidence to recommendation framework was determined to be that desirable consequences clearly outweigh undesirable consequences in most settings (https://www.cdc.gov/vaccines/acip/recs/grade/table-refs.html).

## Recommendation for Hepatitis A Vaccine for Persons Experiencing Homelessness

All persons aged 1 year and older experiencing homelessness should be routinely immunized against hepatitis A (Box 1). Routine vaccination consists of a 2-dose schedule or a 3-dose schedule when combined hepatitis A and B vaccine is administered.

BOX 1 **Recommendations for routine preexposure use of hepatitis A vaccine — Advisory Committee on Immunization Practices**

- All children at age 12–23 months.
- Persons traveling to or working in countries that have high or intermediate HAV endemicity.
- Persons who anticipate close contact with an international adoptee from a country of high or interme­diate endemicity during the first 60 days following arrival of the adoptee in the United States.
- Men who have sex with men.
- Users of injection and noninjection drugs.
- Persons with chronic liver disease.
- Persons with clotting factor disorders.
- Persons who work with HAV-infected primates or with HAV in a research laboratory setting.
- Persons experiencing homelessness.
- Anyone wishing to obtain immunity.

**Sources:** CDC. Prevention of hepatitis A through active or passive immunization: recommendations of the Advisory Committee on Immunization Practices. MMWR Recomm Rep 2006;55(No. RR-7).

CDC. Updated recommendations from the Advisory Committee on Immunization Practices (ACIP) for use of hepatitis A vaccine in close contacts of newly arriving international adoptees. MMWR Morb Mortal Wkly Rep 2009;58:1006–7.

Nelson NP, Link-Gelles R, Hofmeister MG, et al. Update: recommendations of the Advisory Committee on Immunization Practices for use of hepatitis a vaccine for postexposure prophylaxis and for preexposure prophylaxis for international travel. MMWR Morb Mortal Wkly Rep 2018;67:1216–20.

## Clinical Considerations

Concern about loss to follow-up before HepA vaccine series completion should not be a deterrent to initiating the vaccine series in persons experiencing homelessness. One dose of HepA vaccine provides personal protection and can contribute to herd immunity, although long-term protection might be suboptimal (*10*).

Multiple definitions of homelessness have been published in the United States; however, the definitions are similar in content. The U.S. Department of Health and Human Services definition is used for the purpose of this recommendation (Box 2). Because of the difficulty distinguishing the type of homelessness a person is experiencing (e.g., sheltered versus unsheltered) and the associated risks for HAV infection, all persons experiencing homelessness should routinely receive HepA vaccine.

BOX 2 **Homeless definition: U.S. Department of Health and Human Services**

A homeless person is defined as an individual

- who lacks housing (without regard to whether the individual is a member of a family), including an individual whose primary residence during the night is a supervised public or private facility (e.g., shelter) that provides temporary living accommodations and an individual who is a resident in transitional housing;
- without permanent housing who may live on the streets; stay in a shelter, mission, single-room occupancy facility, abandoned building or vehicle; or in any other unstable or nonpermanent situation;
- who is “doubled up,” a term that refers to a situation where individuals are unable to maintain their housing situation and are forced to stay with a series of friends and/or extended family members.

In addition, previously homeless individuals who are to be released from a prison or a hospital may be considered homeless if they do not have a stable housing situation to which they can return. A recognition of the instability of an individual’s living arrangements is critical to the definition of homelessness.

**Sources:** National Health Care for the Homeless Council. https://www.nhchc.org/faq/official-definition-homelessness/.

U.S. Department of Health and Human Services [Section 330 of the Public Health Service Act (42 U.S.C., 254b)].

HRSA/Bureau of Primary Health Care, Program Assistance Letter 99–12, Health Care for the Homeless Principles of Practice.

## Rationale for Recommendation

**Advantages of HepA vaccine for persons experiencing homelessness.** Persons experiencing homelessness might have difficulty implementing recommended nonvaccine strategies to protect themselves from exposure (e.g., access to clean toilet facilities, regular handwashing, and avoidance of crowded living conditions). For this reason, vaccination is the most reliable protection from HAV infection for persons experiencing homelessness. HepA vaccination of persons experiencing homelessness will provide individual protection and increase herd immunity over time, reducing the risk of large-scale, person-to-person outbreaks in this population. The recommendation facilitates routine HepA vaccination of persons experiencing homelessness through facilities that already provide health care services for the homeless population.

**Summary**

**What is already known about this topic?**

Hepatitis A (HepA) vaccine is highly safe and effective, and a complete HepA vaccine series provides long-term protection against hepatitis A virus (HAV) infection. Person-to-person HAV outbreaks among persons using drugs or experiencing homelessness are widespread and ongoing.

**What is added by this report?**

All persons aged ≥1 year experiencing homelessness should be routinely immunized against HAV. Vaccination of homeless persons facilitates integration of HepA vaccine into routine preventive services.

**What are the implications for public health practice?**

HepA vaccination of homeless persons would improve protection of persons at increased risk of exposure to HAV and complications of hepatitis A disease and reduce the risk for large-scale outbreaks by increasing immunity to HAV among homeless persons living in congregate settings where HAV can spread readily.

## Footnotes

* Recommendations for routine use of vaccines in children and adolescents are developed by ACIP, a federal advisory committee chartered to provide expert external advice and guidance to the CDC Director on use of vaccines and related agents for the control of vaccine-preventable diseases in the civilian population of the United States. Recommendations for routine use of vaccines in children and adolescents are harmonized to the greatest extent possible with recommendations made by the American Academy of Pediatrics, the American Academy of Family Physicians (AAFP), and the American College of Obstetricians and Gynecologists (ACOG). Recommendations for routine use of vaccines in adults are harmonized with recommendations of AAFP, ACOG, the American College of Physicians (ACP), and the American College of Nurse-Midwives. ACIP recommendations approved by the CDC Director become agency guidelines on the date published in the *Morbidity and Mortality Weekly Report*. Additional information about ACIP is available at https://www.cdc.gov/vaccines/acip.

† https://www.cdc.gov/hepatitis/outbreaks/2017March-HepatitisA.htm.

§ The ACIP Hepatitis Vaccines Work Group comprises professionals from academic medicine (family medicine, internal medicine, pediatrics, obstetrics, infectious disease, occupational health, and preventive medicine specialists), federal and state public health entities, and medical societies.

¶ In preparation for ACIP deliberation, the scientific literature was searched using PubMed, Medline and EMBASE databases for reports published from January 1, 2000, through April 25, 2018. Search terms excluded studies in nonhumans. Studies were also excluded if they were published earlier than 2000, included only vaccines not licensed in the United States, did not address the population of interest (homeless) or if relevant data could not be extracted. There were no language restrictions on initial searches and articles from any country were included.

** Eleven members voted in favor, with none opposed, none abstained, and none recused.
